# Neuromyelitis Optica: A Peek Into the Brain Through the Eyes

**DOI:** 10.7759/cureus.67408

**Published:** 2024-08-21

**Authors:** Priyanka S Aher, Deepaswi Bhavsar, Sumit Toshniwal, Riddhi A Bora, Rohan Jagadish

**Affiliations:** 1 Ophthalmology, Dr. D. Y. Patil Medical College and Hospital, Pune, IND; 2 Ophthalmology, Dr. D. Y. Patil Medical College, Hospital and Research Center, Pune, IND

**Keywords:** sjogrens syndrome, demyelinating syndrome, optic neuritis, nmo-igg antibodies, neuromyelitis optica spectrum disorder (nmosd)

## Abstract

A rare demyelinating syndrome, known as neuromyelitis optica, is characterized by optic neuritis and transverse myelitis. A 27-year-old female presented to the eye department, with complaints of sudden progressive diminution of vision in both eyes (left eye more than right eye), acutely not being able to move both her eyes, double vision on lateral gazes with chronic dryness and irritation. Examination revealed restricted extraocular movements in all gazes. Magnetic resonance imaging of orbit showed retrobulbar optic neuritis. Blood tests revealed positive neuromyelitis optica myelin oligodendrocyte glycoprotein antibodies after which diagnosis of neuromyelitis optica was made.

## Introduction

A rare demyelinating syndrome, known as neuromyelitis optica (NMO), is characterized by optic neuritis and transverse myelitis. First considered as a variant of multiple sclerosis (MS), it is now considered as an entirely different condition. Identification of aquaporin-4 (AQP4) antibody in the serum has a very high specificity [1]. Thus, the target of the NMO antibody is the AQP4. Neutrophils and eosinophils play a major contributory role in the tissue damage. NMO and spectrum disorders (NMOSD), also known as Devic disease, constitute 13.9% of all demyelinating disorders, with a prevalence of 2.6/100,000 [2].

One important subset of NMO spectrum disorder is those patients who are known to test positive for myelin oligodendrocyte glycoprotein (MOG) antibody. As with NMO, it includes optic neuritis, myelitis, and brainstem syndromes, though there are subtle differences between the two conditions. Optic neuritis in MOG antibody-associated disease often involves a long segment of the optic nerve and can present with disc edema, with myelitis often involving lower segments of the spinal cord than in AQP4 antibody disorder. 

NMOSD is a relapsing condition consisting of optic neuritis, myelitis, or area postrema syndrome. Optic neuritis in NMO is severe, commonly bilateral, and the recovery is not always complete. NMO can present as acute isolated optic neuritis at initial presentation [3]. Intractable hiccups and nausea as initial presenting symptoms have been reported in 43% of cases [4] of NMO patients, positive for anti-AQP4/NMO-IgG antibodies [5-7]. There is a “rim and rosette” pattern of immune complexes in the NMO lesion which is a result of complement activation at the site of the AQP4 protein.

Longitudinally extensive myelitis (LETM) is another hallmark of NMO. LETM is lesions including three or more vertebral segments. LETM or more limited TM starting in the cervical spine and reaching into the brainstem may lead to respiratory failure and/or persistent intractable hiccups and nausea, both of which are regarded as typical for NMO [8,9]. Myelitides may involve motor, sensory, and/or bowel and bladder functions. Brainstem symptoms present as area postrema syndrome which includes intractable nausea and vomiting, hiccupping. Other symptoms include double vision, dysphagia, and respiratory compromise.

Key diagnostic test of NMO antibody has a sensitivity of 70%-80% and specificity close to 100%. Patients with NMO often have antibodies positive for other conditions like Sjogren's syndrome or Lupus, suggesting two coexisting autoimmune syndromes. Cerebrospinal fluid in NMO shows mile elevation in white blood cells, and the presence of oligoclonal bands in 10% of cases [10].

The treatment approach in NMOSD and MOG antibody disease is almost identical which includes treatment of relapse, prevention of relapse, and treatment of chronic symptoms. Attack of NMOSD is treated promptly and aggressively with Intravenous methylprednisolone for a period of five days followed by prednisone tapering. Plasmapheresis or intravenous immunoglobulin may be used in some unresponsive patients with relapses who have incompletely recovered after steroid treatment. Plasmapheresis has been shown to bring a better outcome when used earlier in the course [11].

## Case presentation

A 27-year-old female, not a known case of any systemic illness, who was admitted to the neurology ward was referred to the eye OPD with not being able to move both eyes, associated with a binocular double vision on trying to move her eyes and diminution of vision in the left eye more than right eye for four days. These symptoms were sudden and progressive, not associated with pain. She also complained of chronic irritation and dryness in her eyes. She was admitted to the neurology ward in view of right-sided weakness, difficulty in walking, and numbness progressing over four days, with difficulty in speaking, and excessive sleeping associated with flexor spasms. She also had tingling sensations intermittently over her limbs. She had no bowel and bladder complaints, but she did complain of frequent hiccups. She had similar complaints three months back for which she had taken a course of intravenous steroids for three days, followed by oral steroids after which her symptoms had partially resolved. There was no complaint of difficulty in closing eyelids, flashes, or floaters.

**Figure 1:**
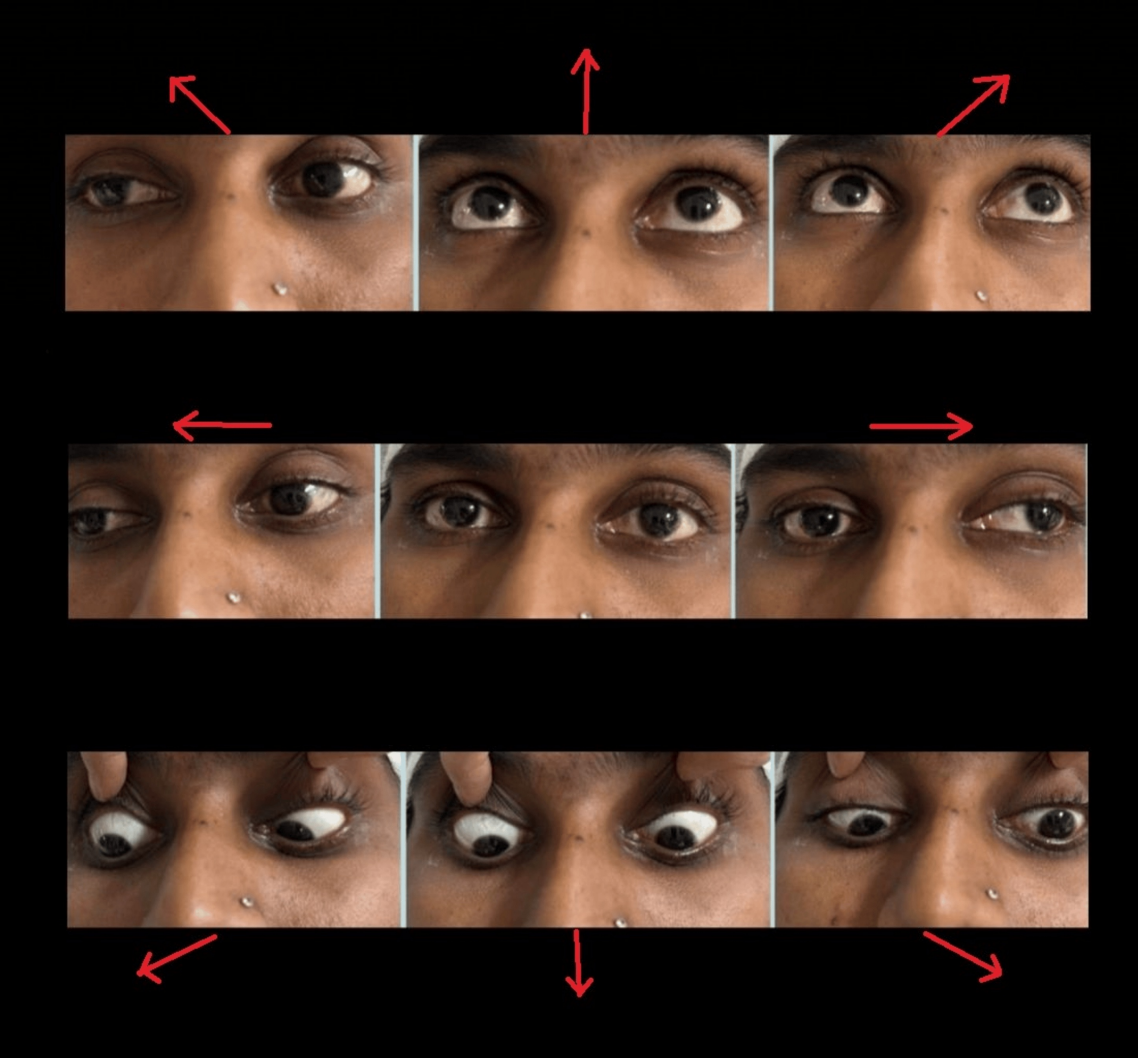
Limited extraocular movements of both the eyes in all the directions (arrows).

On evaluation, the best corrected visual acuity in the right eye was 6/60, and in the left eye was 1/60 recorded on Snellen’s chart. Color vision was impaired in the left eye. Extraocular movements in both eyes were limited in all quadrants as shown in Figure 1.

On anterior segment examination, there was mild diffuse congestion in both eyes with grade 2 relative afferent pupillary defect (RAPD) in the left eye. Both eye fundus showed no abnormality on indirect ophthalmoscope and slit lamp examination. Power was decreased in all four limbs (4/5). Sensory examination showed no abnormality. Cerebellar examination showed ataxia.

**Figure 2:**
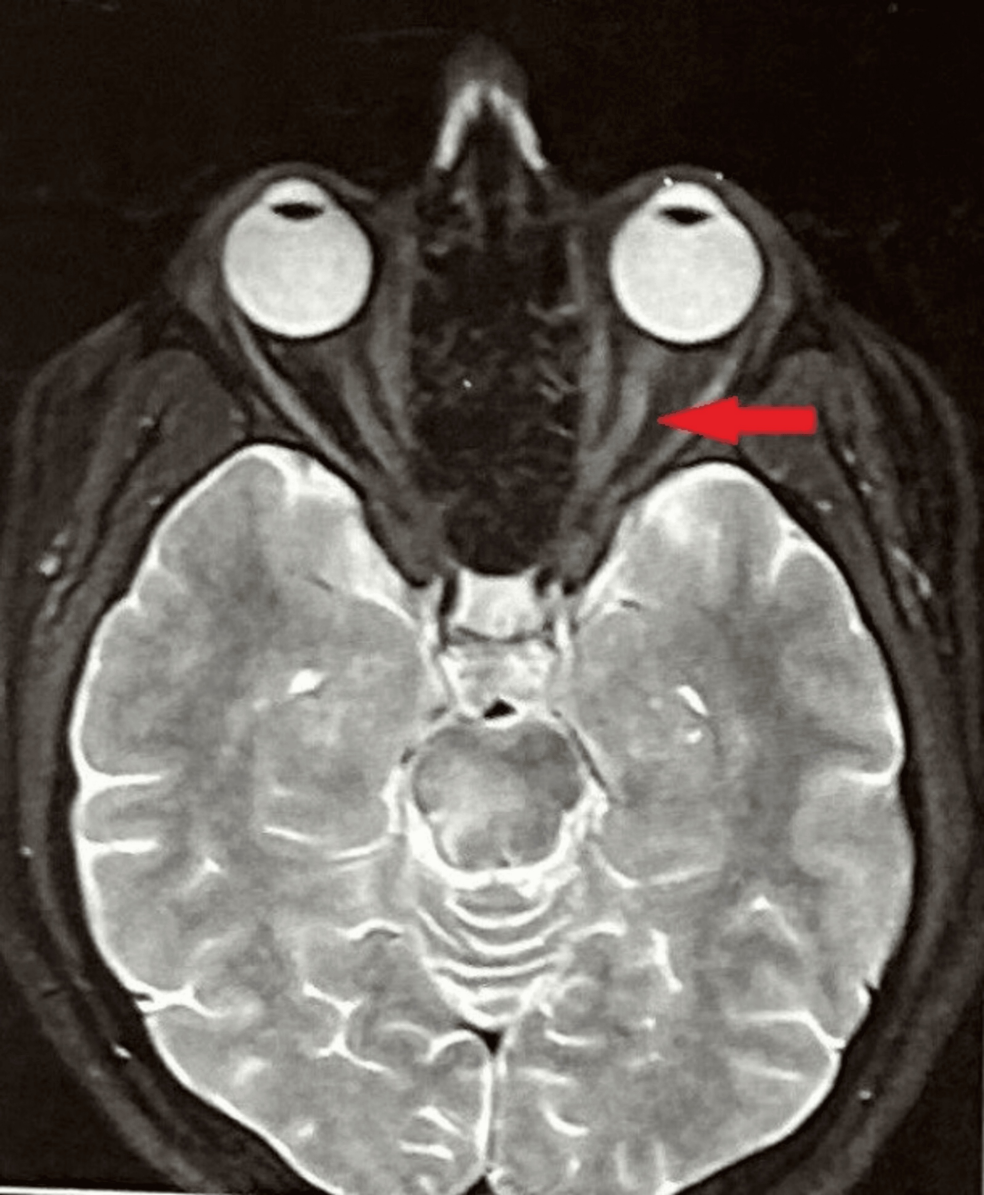
MRI showing left optic neuritis.

Magnetic resonance imaging (MRI) of the orbit revealed edematous left optic nerve. T2/FLAIR showed a hyperintense signal suggestive of left optic neuritis as shown in Figure 2.

MRI spine was suggestive of paraspinal muscle spasm with mild posterior bulge at L5-S1 levels indenting the thecal sac. White blood cell count revealed increased ratio of neutrophils with 79% (normal range - 40%-60%). The rest of the blood counts were within normal range. The visual evoked potential test showed pattern reversal with prolonged P100 wave latency suggestive of anterior pathway defect.

The patient was provisionally diagnosed with MS and started on 1 g intravenous methylprednisolone for three days. As there was no sufficient improvement symptomatically, more investigations were done which revealed positive NMO MOG antibodies in the blood, and ACE levels were elevated. P-ANCA, C-ANCA were negative. The cerebrospinal fluid examination had the presence of oligoclonal antibodies. She had to be started on plasmapheresis alternate day five cycles. She was also given 1 g of rituximab over 24 hours prior to discharge. The patient showed improvement symptomatically. She was able to walk without support. Her vision improved to 6/60 in the left eye. She was discharged on oral prednisolone 40 mg as maintenance therapy for one month and called for one monthly follow-up.

## Discussion

NMO is an antibody-mediated disease involving the central nervous system. this disease was initially thought to be a severe form of optico-spinal MS [12]. with the identification of AQP4 antibodies in patients with NMO, it can be differentiated from MS. These antibodies are present in other tissues including collecting ducts of the kidneys, parietal cells of the stomach, secretory glands, airways as well as skeletal muscles but they are spared in this disease due to local complement inhibitors which are absent in the brain [13,14].

In the above-mentioned case, the patient presented with symptoms that included diminution of vision in both eyes, limited restriction of movement of both eyes in all gazes, and weakness of limbs. These symptoms are present in MS as well, the patient was initially diagnosed with MS. The presence of AQP4 antibodies and oligoclonal antibodies in CSF confirmed the diagnosis of this patient as NMOSD. In this patient, she responded better with plasmapheresis along with steroids instead of steroids alone and had to be kept on long-term oral steroids. Rituximab was given as an immunosuppressive therapy to prevent relapses, and the patient had to be kept on regular follow-up. 

## Conclusions

NMO can present as an acute onset of limitation of extraocular movements and a decrease in visual acuity. Detailed ophthalmological as well as neurological evaluation has to be done in such patients. MS is the most common diagnosis suspected in patients presenting with the above-mentioned complaints. The presence of AQP4 antibodies in blood, the presence of oligoclonal antibodies on cerebrospinal fluid examination, and correlation with clinical features were the most important diagnostic criteria in this patient. Symptoms improve with treatment, majorly with plasmapheresis and intravenous corticosteroids, but it is a recurrent disease, and the patient has to be kept on long-term oral steroids with regular follow-up and immediate response in case of recurrent acute episodes.
